# Authors’ reply to ‘Multiple comparisons controversies are about context and costs, not frequentism versus Bayesianism’

**DOI:** 10.1007/s10654-019-00566-7

**Published:** 2019-10-04

**Authors:** Arvid Sjölander, Stijn Vansteelandt

**Affiliations:** 1 Karolinska Institutet, Nobels väg 12 A, 171 77 Stockholm, Sweden; 2 Ghent University, Krijgslaan 281, S9, 9000 Ghent, Belgium

## Introduction

We thank Sander Greenland and Albert Hofman (hereafter abbreviated GH) for their comments [1] on our paper [2], and the Editor for giving us the opportunity to respond. We have made an effort to understand the foundations for GH’s critique, hoping that we don’t misrepresent them in our response below. We first summarize and add clarity to the main points of our paper.

## Our main points

In our experience, multiple testing is surrounded by confusion. This confusion seems to stem primarily from a difficulty to distinguish between relevant and irrelevant multiplicity adjustments. In our paper we have argued that

- the frequentist framework is unable to distinguish between relevant and irrelevant multiplicity adjustments; from the frequentist perspective all possible collections of tests seem equally valid to adjust for.
- the Bayesian framework, by using posterior probabilities as a basis for inference, offers a clear and coherent distinction. Within this framework it is relevant to adjust for those tests that are associated with the test under consideration. Here, we defined two tests to be associated if either the hypotheses, or the data sets, of the tests are associated. Within the Bayesian framework, the association between hypotheses is encoded by joint prior distributions on these.

We have shown that this formality of the Bayesian framework helps to reason about the set of relevant adjustments in the light of background context, which we expressed by means of directed acyclic graphs. We noted, however, that it may be hard to specify joint prior distributions, and that the set of relevant adjustments may be extremely large. Thus, for practical purposes we proposed a compromise, where the formal statistical analysis is done within the standard frequentist framework (e.g. by computing *p* values or confidence intervals), and the adjustment for multiplicity is done informally, by reasoning qualitatively about the association of hypotheses. In particular, we have recommended surveying the available evidence and making it available as part of the ‘Discussion’ section of applied epidemiological papers. One step further would be to borrow information across tests or comparisons. In that sense, we view GH’s favored hierarchical modeling approach as being in line with (and a further formalization of) our recommendations.

Our reasoning on directed acyclic graphs has further led us to draw two conclusions that may not seem immediately intuitive. First: when testing two hypotheses on related data set, the Bayesian framework generally requires adjustment of the first test for the second test, even if the two hypotheses are unrelated. This is so because the test statistic of the first hypothesis is a collider between the first hypothesis and the test statistic of the second test. Second: when doing a fishing expedition on unrelated hypotheses and data sets, there is no need to make any multiplicity adjustment within the Bayesian framework, since the posterior for any of the hypothesis does not, in this case, depend on the test statistics of the other tests or on the selection mechanism induced by the data fishing. We would like to emphasize that this is only so provided that a correct Bayesian analysis is considered, and that standard frequentist measures (e.g. *p* values) are not necessarily justified under an (informal) frequentist analysis.

## GH’s critique

As we understand it, GH’s critique mainly concerns five issues; frequentism versus Bayesianism, formal versus informal adjustment, information summary versus decision making, hierarchical models, and context and causality.

### Frequentism versus Bayesianism

GH argue that our dichotomization between ‘frequentist’ and ‘Bayesian’ frameworks is overly simplistic. He writes: ‘There is far more in our toolkit than the extremes of frequentist and Bayesian’, and ‘There is no singular frequentist or Bayesian philosophy or methodology any more than there is just one form of (say) Christianity’.

We agree with GH on this point. However, the fact that there is a strong heterogeneity within Christianity does not imply that anything would reasonably qualify as Christian, or that it would be meaningless to make comparisons between Christianity and, say, Islam. In our paper we (somewhat implicitly) defined ‘frequentism’ as the collection of methods that treat parameters as fixed, and use the distribution of data, given parameters, as basis for inference. We defined ‘Bayesianism’ as the collections of methods that treat parameters as random, and use the distribution of the parameters, given data, as basis for inference. Clearly, there are many variations of and hybrids between these, such as mixed effects models, empirical Bayesianism etc. However, despite this plethora of methods, epidemiological practice today is absolutely dominated by the extreme frequentist end of the spectrum, where all parameters are treated as fixed and inference is solely based on the distribution of data, given parameters, as in Bygren et al. [3]. There may be acceptable reasons for this conformity, such as a genuine consensus that ‘extreme frequentism’ is, by and large, a reasonable scientific attitude. There may also be less acceptable reasons, such as tradition or ignorance about the alternatives. The underlying reasons do not matter much for our discussion though; what matters is that most epidemiologists think and act within the (extreme) frequentist framework, as we have defined it, and that this framework obscures discussions around multiplicity adjustments. In particular, regardless of the above dichotomization, the key point is that the posterior distribution provides a formal tool for distinguishing between relevant and irrelevant multiplicity adjustments.

### Formal versus informal adjustment

GH tentatively accept our proposal for informal adjustment in studies where ‘the analysis targets only one focused research question represented by a few closely related statistical hypotheses or parameters, and all analyses and estimates are reported with equal emphasis and detail’. However, they reject the proposal for studies where ‘there are several interdependent parameters or hypotheses in the analysis’, and for studies that are ‘highly exploratory, aiming to inform decisions about which of many weak possibilities to pursue with focused efforts’. For such scenarios they advocate formal adjustments with hierarchical models, as illustrated by Greenland [4] and Witte et al. [5].

We agree with GH that our proposed informal adjustment may not always be appropriate, and that there are situations where a more formal approach may be preferable. However, we note that a formal adjustment may quickly become unmanageable. For instance, real epidemiological studies often contain a large number of ‘primary analyses’, ‘secondary analyses’, ‘sensitivity analyses’ and so forth, not to mention all other aspects of the analyses that entail more or less implicit multiplicity issues, such as model selection, variable selection etc. Furthermore, some of these may be presented in the main text of the paper, whereas some may only be presented in online supplementary material, or just referred to as ‘carried out but not shown’. To put all these into one hierarchical model adds complexity and may not be realistic. Even worse, as shown in our paper the Bayesian framework suggests that adjustment should in principle be made for ‘all other tests in the world that are associated with the test under consideration’, which is clearly not possible with a single unifying model. Thus, even though formal adjustment may be possible, or even desirable, in contextually well-informed settings such as those in Greenland [4] and Witte et al. [5], without considerable effort to formalize this information accurately one will reach a point where formal treatment must give way to, or at least be complemented by, more informal considerations. In our opinion, one can come a long way in reporting and visualising the available evidence, as in a forest plot. This is useful regardless of whether the evidence is also summarised, pooled or borrowed across results (as in hierarchical models), each of which tend to give more optimistic impressions of the available evidence (by extracting additional information from a priori assumptions and/or other study results).

### Information summary versus decision making

GH emphasize the distinction between information/evidence summary and decision making. He writes: ‘Unfortunately, much of the statistical literature (including SV) fails to distinguish between these two tasks.’ He further emphasizes the role of loss functions for multiple testing: ‘Loss functions are central to justifying any conclusive statement about a relation. Whether a claim is of no effect, or harm, or benefit, it entails an implicit belief that the conclusion is justified because the cost of being mistaken (which is always a risk) is less than the cost of being inconclusive or incorrectly concluding something else.’

We agree with GH that the distinction between information/evidence summary and decision making is important, and that loss functions are essential for the latter. However, in our experience the confusion surrounding multiple testing is almost exclusively related to information/evidence summary, and this was also the sole focus on our paper. We believe that this is pretty clear from how the paper was written. For instance, we wrote [just below equation (2)]: ‘...if the posterior probability is greater than 0.5, then the alternative hypothesis is more probable than the null hypothesis, so it would be rational to *believe* in the alternative, i.e. to estimate that $\theta _f$ is equal to 1.’ (Emphasis added). Thus, our paper is considered with beliefs per se, not about acting on these beliefs. In particular, we did not discuss how to make decisions (based on posterior probabilities) in light of the costs of decision errors.

To put this in the context of Bygren et al. [3], consider Table 1 from our paper. The controversy around this table can be formulated like this: ‘Given the seven non-significant *p* values in this table, is it rational to *believe* that epigenetic effects are truly present in the stratum of paternal grandmother/female grandchild, where a significant *p* value was found?’ This question is about evidence, not about decisions. Thus, our answer to this question should not depend on what potential (e.g. political, medical etc) consequences it may have to ‘formally declare’ (e.g. in the title of a scientific paper, as in Bygren et al. [3]) that we do or do not believe in epigenetic effects.

### Hierarchical models

GH strongly advocate the use of hierarchical models for handling multiplicity problems. They say: ‘hierarchical modeling provides a coherent framework for fine-tuning models to maximize valid information (signals) in the compressed data while minimizing random artefacts (noise)’.

We agree that hierarchical models are often useful, but we believe that they only go half-way in providing solutions to the more logical/philosophical problems with multiple testing. To see why, consider the paper by Witte et al. [5], which GH put forward as a prominent example of a hierarchical model analysis. In this paper, the authors studied the associations between 12 food items (e.g. cauliflower, tomato) and breast cancer. They first used a conventional logistic regression on the form

$$\begin{aligned} {\text {logit}}\{p(Y=1|{\mathbf {X}})\}=\alpha +\beta {\mathbf {X}}, \end{aligned}$$

where *Y* is a cancer indicator and ${\mathbf {X}}$ is a vector of consumed food items. Witte et al. [5] included covariates (e.g. age, body mass index) in the model as well; for simplicity we ignore covariates here. The vector of food coefficients $\beta$ is the target parameter. Fitting this model with standard maximum likelihood gave quite disparate estimates, with positive coefficients for some food items, but negative coefficients for other. The authors commented on this by saying ‘a priori we expected all foods to be either inversely associated or not associated with breast cancer owing to their known constituents’ potential anticarcinogenic properties’. They proceeded by fitting a hierarchical model, in which the food coefficients were modeled as

$$\begin{aligned} \beta =\pi {\mathbf {Z}}+\delta , \end{aligned}$$

where $\pi$ is a vector of fixed parameters and $\delta$ is a random error term, assumed to have a normal distribution with mean 0 and fixed assumed variance $\tau ^2$. The matrix ${\mathbf {Z}}$ contains food constituents that may contribute to dietary effects on breast cancer (e.g. glucosinolates, fiber), so that element $z_{ij}$ of $\mathbf {Z}$ is the amount of constituent *j* in food item *i*. When fitting this hierarchical model, the estimated food coefficients were ‘shrunk’ towards each others, which then, presumably, would indicate that a multiplicity adjustment has taken place.

The amount of shrinkage in the estimated food coefficients depends on two things; the relative dimensions of $\pi$ and $\beta$, and the assumed variance of $\delta$. If the dimension of $\pi$ is small, relative to the dimension of $\beta$, then the food coefficients are projected into a smaller subspace, so that there will be large shrinkage. If, in addition, the variance of $\delta$ is small, then the food coefficients are prevented from varying within this subspace, which will further increase the shrinkage. An extreme case occurs when $\pi$ is a scalar and the variance of delta is assumed to be 0, in which case maximal shrinkage occurs.

Now, suppose for pedagogical reasons that there are equally many constituents and food item (i.e. 12). Suppose further that, as it turns out, each food item only contains one constituent, and each constituent only appears in one food item, i.e. that $\mathbf {Z}$ is a diagonal matrix, so that $\beta _i=z_{ij}\pi _i$. In this case, the dimensions of $\beta$ and $\pi$ are identical, and there will be no shrinkage, regardless of the assumed variance of $\delta$. Thus, the researcher is left without any guidance on whether and how to do multiplicity adjustment, since the hierarchical model will not do that for him. If the researcher nevertheless proceeds by using a hierarchical model in this case (or just an ordinary regression model, since the hierarchical model didn’t suggest adjustment anyway), and reports the unadjusted *p* values from the fitted model, then a pure frequentist could launch the same critique as Häggström [6] did against Bygren et al. [3] (‘with so many unadjusted tests, there is a large risk that you have obtained at least one false positive’), without being able to articulate where the poor researcher should have drawn the line between relevant and irrelevant adjustments.

However, by using ‘fully’ Bayesian arguments, the neglect to adjust for multiplicity may be justifiable. If we have no reason to believe that different food items should have similar effects on breast cancer, except if they happen to share any of the 12 constituents that we have listed, and if the ‘residual association’ due to dependent data (represented by the dashed double-headed arrow in our Figure 2) is weak, then it makes perfect sense from a Bayesian perspective to not adjust for multiplicity, since the posterior distribution for each particular food coefficient is roughly unchanged by such adjustment.

### Context and causality

GH emhasize the need for contextual information in multiplicity problems. They write: ‘context immersion, not mathematical statistics, is essential to specify a contextually sensible point between the poor extreme of no adjustment and the absurd and impossible extreme of adjustment for everything. In this regard, *any failure of a method to formally specify the adjustment set is an honest response to a question that cannot be sensibly answered by using abstract, decontextualized statistical rules*’.

We believe that GH, perhaps unintentionally, make a poor caricature of our view here. We have never claimed that context is unimportant, or that contextual thinking can be replaced by automatized rules. On the contrary, we have emphasized (see for instance Section ‘Practical implications’) how utterly important contextual reasoning is in multiplicity problems. What we have claimed though, is that (1) the Bayesian framework offers a principled way of incorporating contextual information whereas the frequentist framework does not, and (2) from the Bayesian perspective, contextual information only matters insofar that it influences the joint prior distributions of parameters/hypotheses.

The second point does not mean that the analyst needs to specify, or make a guess about, joint priors directly. In many situations it would be more natural to derive these from more fundamental relations, either through a formal (e.g. hierarchical) model, or through informal reasoning. For instance, as GH point out, causal considerations are important in many practical problems, even if the research question is non-causal, since underlying causal structures may explain why and to what extent we should consider parameters/hypotheses a priori associated.

Regarding causality, GH make an analogy between multiplicity adjustment and confounding adjustment. They write: ‘to claim that every comparison should be adjusted for every other comparison (even comparisons never carried out by the analyst) is as detached from reality as claiming that every causal analysis of an observed association between two variables must adjust for every conceivable shared cause of the variables going back to start of our universe.’ Presumably, the part about ‘adjusting for every other comparison’ is intended to describe our view, which we again believe is a poor caricature. One of the main messages of our paper is that the Bayesian perspective relieves the analyst from many multiplicity adjustments that may seem intuitively necessary (see Section ‘Data fishing and the intention of the researcher’).

Nevertheless, we do believe that the analogy with confounding adjustment is useful, as it pinpoints the need for a principled framework, as well as the need to distinguish between principles and pragmatic practice. Today, there is an overwhelming consensus that causal diagrams offer a principled framework for confounder selection, and that causal diagrams are immensely helpful to structure thinking about which covariate adjustments are relevant and which are not [7–12]. We believe that the Bayesian framework has a similar pedagogical value for multiple testing problems. However, as GH allude to, if one would meticulously draw a causal diagram for a real epidemiological scenario, aiming to include all covariates that would in principle be relevant to adjust for, one would quickly come to a point where adjustment for these becomes unmanageable, simply because there would be too many. Similarly, we argue in our paper that the Bayesian framework may suggest an extremely large set set of multiplicity adjustments. This does not deprive either tool of its pedagogical value though; what it means is simply that we have to find a reasonable compromise between adjusting for everything and adjusting for nothing, and perhaps also between formal and informal adjustment. However, before this can be done, one needs a principled framework to distinguish between relevant and irrelevant adjustments, so that we at least know what we would do in a ‘perfect world’ not hampered by practical considerations.

## References

[1] Greenland S, Hofman A (2019). Multiple comparisons controversies are about context and costs, not frequentism versus Bayesianism. Eur J Epidemiol.

[2] Sjölander A, Vansteelandt S (2019). Frequentist versus Bayesian approaches to multiple testing. Eur J Epidemiol.

[3] Bygren L, Tinghög P, Carstensen J, Edvinsson S, Kaati G, Pembrey M, Sjöström M (2014). Change in paternal grandmothers early food supply influenced cardiovascular mortality of the female grandchildren. BMC Genet.

[4] Greenland S (1992). A semi-Bayes approach to the analysis of correlated multiple associations, with an application to an occupational cancer-mortality study. Stat Med.

[5] Witte J, Greenland S, Kim L, Arab L (2000). Multilevel modeling in epidemiology with GLIMMIX. Epidemiology.

[6] Häggström O (2016). The need for nuance in the null hypothesis significance testing debate. Educ Psychol Meas.

[7] Hernan MA, Hernandez-Diaz S, Werler MM, Mitchell AA (2002). Causal knowledge as a prerequisite for confounding evaluation: an application to birth defects epidemiology. Am J Epidemiol.

[8] Jewell NP (2004). Statistics for epidemiology.

[9] Glymour MM, Oakes JM, Kaufman JS (2006). Using causal diagrams to understand common problems in social epidemiology. Methods in social epidemiology.

[10] Rothman KJ, Greenland S, Lash TL (2008). Modern epidemiology.

[11] Pearl J (2009). Causality: models, reasoning, and inference.

[12] Pearl J, Mackenzie D. The book of why: the new science of cause and effect. Basic Books; 2018.

